# Challenges to Cardiac Rehabilitation Post Coronary Artery Bypass Grafting: A Qualitative Study in Pune

**DOI:** 10.7759/cureus.35755

**Published:** 2023-03-04

**Authors:** Nirankush Borah, Jitendra S Bhawalkar, Hetal Rathod, Vallari Jadav, Shweta Gangurde, Johnson S

**Affiliations:** 1 Community Medicine, Dr. D. Y. (Dnyandeo Yashwantrao) Patil Medical College Hospital and Research Centre, Dr. D. Y. Patil Vidyapeeth, Pune, IND

**Keywords:** diet, follow-up, exercise, barrier, cabg, cardiac rehabilitation

## Abstract

Background

Adherence to cardiac rehabilitation (CR) regimen is crucial in the post-coronary artery bypass grafting (CABG) period. Cardiac rehabilitation involves various lifestyle changes, including diet and exercise, regular follow-up in OPD, and implementing secondary prevention recommendations. This study aims to understand the challenges to CR post-CABG.

Methodology

Seven in-depth interviews using an interview guide were carried out in the outpatient section of the cardiothoracic vascular surgery (CTVS) department of a tertiary health care facility in Pune, India. A purposive sampling technique was followed.

Results

The qualitative study revealed that dietary restrictions were difficult to follow, that some patients could not quit smoking even after surgery, and that transportation costs were an issue regarding CR follow-up. The majority of the participants appreciated the importance of regular exercise in their post-surgery life.

Conclusion

Despite the hurdles, the majority of participants stated that the fact that CABG is a major treatment pushed them to attend routine CR follow-ups and adhere to the CR regimen. A solid CR team was one of the study's strengths, and the team established a routine of telephonic reminders for regular follow-up, which resulted in lower rates of loss to follow-up.

## Introduction

Ischaemic heart disease (IHD) affects roughly 126 million people (1,655 per 100,000), or around 1.72% of the world’s population. IHD, which has already claimed the lives of nine million people worldwide, affects men more frequently than women. The onset typically begins in a person’s fourth decade and intensifies with age [1]. Implementing cardiac rehabilitation (CR) is a valuable and cost-effective technique in improving the effectiveness of post-cardiovascular disease (CVD) care. CR is comprised of a series of activities designed to improve the patient’s physical and mental health, lifestyle, reduce risk factors that lead to adaptive behaviours, and maintain optimal daily functioning [2]. Very few qualitative research studies on adherence to CR have been conducted in India. This study was undertaken in Pune, India, to explore the challenges to CR post-coronary artery bypass grafting (CABG).

## Materials and methods

The study was undertaken at a tertiary care hospital in Pimpri-Chinchwad municipal corporation, Pune. The cardio-thoracic vascular surgery (CTVS) department of this hospital provides an in-house comprehensive cardiac rehabilitation program with a strong emphasis on physical activity, diet, and follow-up in the postoperative period after coronary artery bypass graft surgery. A tailored lifestyle modification regimen, home exercises, and counseling are advised as per the respective patient’s characteristics.

Using the purposive sampling technique, 12 patients who underwent CABG and presented to the outpatient department of CTVS as a part of their post-surgery follow-up were approached for face-to-face interviews. Institute ethics committee clearance was obtained. Seven participants agreed to an in-depth interview conducted using an interview guide (see Appendix) at a tertiary healthcare hospital in Pune, Maharashtra. Interviews were conducted in Marathi/Hindi for the convenience of the participants and were audio recorded after receiving consent. The interviews were then transcribed and translated into English. Formal introductions included greetings and icebreakers, which were then followed by the main topic of the conversation. Nonverbal cues and indications, including gestures, nodding, and smiling were also recorded. The interview lasted until there was no longer any new information to be obtained. The average time per interview was approximately 30 minutes per participant. The participants were allowed to ask questions or request clarifications after the in-depth interviews, and their concerns were addressed. The note-takers provided verbatim and accurate notes. A thematic analysis was carried out.

## Results

An attempt has been made to understand the challenges to CR post-CABG. Out of the seven participants who agreed to the interview, six were males and one was female. The youngest participant was 27 years old. The following seven themes emerged from the interviews (as shown in Table 1):

**Table 1:** Themes that emerged from the responses of the study participants CABG: Coronary artery bypass grafting

Themes	Characteristics
Dietary Restrictions	Restrictions on eating outside food, with occasional cravings for it
	Eating less spicy food
	Now accustomed to eating less spicy food
	Dietary restrictions are not easy
Exercise/Physical Activity	Walking on farms
	Family responsibilities
	Forget to do spirometer exercises
	Can’t continue for life
Addiction	Can’t quit smoking, family doesn’t understand
	Heavy alcoholic, quit drinking after surgery
	Occasionally consumes alcohol
	Stopped smoking
Regular Follow-up in OPD	Doesn’t feel follow-ups are a burden; feels happy to meet doctor
	Private transport is a financial burden
	No problem in regular follow-ups
Financial Constraints	Medications are costly
	Participant has stopped working, wife goes to work now
	Don’t want to be a burden on anyone, participant has resumed working
Stress after CABG	Fearful and restless
	Has become calmer
	No stress, has a history of multiple CABG in family
Others	Concerned about decrease in libido

Dietary restrictions

Dietary changes primarily involved the consumption of fewer spicy and fatty foods. The dietary adjustments prohibited participants from eating outside food. The majority of them believed that dietary adjustments were necessary for their health. According to another participant, the improvements can be difficult to implement. One individual claimed that the reminders during routine follow-ups in OPD assisted him in maintaining the dietary changes. Another participant claimed that she has now adapted to the dietary changes after implementing them.

P1 - “I have been prohibited from eating outside food. I only eat homemade food now. As my doctor has advised, I can eat non-veg food like chicken, fish, and eggs but I eat only boiled eggs without yolk.”

P2 - “Dietary intake care is very important. Rice and lentils don’t cost much.”

P3 - “Ata savay padli” [Now I am used to it]. On enquiring how dietary changes after CABG made her feel, the participant responded that she was accustomed to eating less spicy food. “If I eat spicy food, I get a burning sensation in my chest. Now I consume spicy food sometimes for my taste buds. I also take pickles occasionally to add taste.” [OJ1]

P5 - “Aasan nahi hai. Kya bole, beemar pad gae to paalne to padega” [The dietary restrictions are not easy. What do I say? I have fallen sick, so I have to follow the dietary restrictions].

P6 - “I used to eat a lot of outside food earlier, but now I have stopped it completely.” When asked whether he has urges to eat outside food, the participant replied, “Mann to aata hai bahar ke khaane par, par ab kya karenge” [I feel like eating outside food, but can’t help it].

P7 - “Shuru mein aisa lagta tha apun control nahi karenge, sab khana kha lenge. Lekin aapka jo follow up lete time har baar wahi soochna milti haina, ussae bohot control ho gaya mujhe” [Initially I used to think that I can’t control and will eat everything. During follow-up, same information was conveyed which helped me improve my dietary habits].

Physical activity - An actual long-term commitment

One of the crucial components in CR involves regular physical activity.

While the majority of the participants started engaging in regular physical activities after CABG, a few believed that their routine daily activities constituted enough physical activity. As Participant 2 stated, “I don’t go for long walks, I just walk while I am on the farm.” Overall, they all appreciated the importance of regular exercise in their post-surgery life. Others struggled with exercises involving a spirometer. One of them said that he found it challenging in maintaining the long-term exercise regimen.

P1 - “Meri bacchi ki delivery ho gai toh mujhe 2-2 naati sambhalna padta hai, 2 mahine toh hue fookne wali machine band kiya. Ek plastic ka machine to meri naati ne fod diya” [My daughter delivered a few days ago and I have to take care of two grandchildren. So I have stopped doing spirometry exercises for two months. One of my grandchildren broke that machine. Also, recently I have been unable to walk for five kilometres].

P3 - “Vyayam nahi kela tar nasa tasach rantil, vyayam kela tar nasa moklya rahatil” [Sometimes I forget to do spirometry exercises. If I do physical exercise, it makes my nerves relaxed. I will try to do exercise as long as I can do it, we have to take care of our life].

P4 - “I don’t think I will be able to continue exercising for life. I will do it as much as I can tolerate.”

P6 - “Chalne se khoon patla hota hai, isliye chalna zaroori ha” [Walking helps in thinning of blood, it is very important. I work as a security guard hence I keep walking during my duty hours also], answered one 27-year-old participant who underwent CABG in June 2022. He also added further, “I stopped doing spirometry exercises because I completely forgot about it.”

Take on addiction - A stubborn habit

Smoking and alcohol intake are known to contribute to coronary artery disease (CAD). The majority of participants had quit smoking and alcohol. One of the participants responded that he could not quit smoking and his family did not understand that he had urges to smoke to clear his bowels. Another participant accepted the fact that his habit of smoking led him to the hospital and because of this, he had no choice but to give up the smoking habit.

P1 - “I used to smoke earlier. This habit landed me over here. Now, however, I’ve quit following the surgery.”

P4 - “I have not quit smoking. I also take alcohol. I have urges to smoke. I can’t go to the washroom in the morning without smoking. My family doesn’t understand this. They keep shouting at me to quit smoking.”

P5 - “I used to be a heavy drinker and smoker, but I have stopped smoking now. But I occasionally take alcohol.”

P6 - “I used to be a heavy alcoholic. Now I have stopped.” “Itna bada operation hone ke baad kaise mann karega?” [After undergoing such a major surgery, how would I feel to drink?].

Follow-up for cardiac rehabilitation

An important part of CR involves visits to the cardiac OPD and consultation with cardiovascular surgeons. Sometimes, the patient has to travel long distances to reach the tertiary health care centres and may find this burdensome. One individual responded that using private transportation was expensive, hence he had to switch to the public transport system so as not to miss follow-ups in the OPD. Another participant mentioned that meeting with the doctor for follow-ups makes him happy, hence, the transportation cost was not a barrier for him.

P2 - “I have to spend nearly 13,000 to 14,000 INR if I use a private vehicle for transportation. Now I have started using public transport to attend Cardiac Rehabilitation follow-ups.”

P4 - “I don’t feel that follow-ups are a burden. In fact, I feel happy to meet the doctor, so that I can share my problems.”

P6 - “I have no problems in regular follow-ups.” “Abhi itna bada operation hua toh kaun bhoolega, apni jaan ki fikar to sabko hai” [One cannot forget such a big surgery, everyone worries about their life].

Need for a cardiac rehabilitation group

Respondents pointed out that they valued the social aspect of CR. One of the most significant benefits of participating in the group-based CR programme was the opportunity to engage and converse with individuals who have undergone the same surgery, and thus, can relate to their concerns and problems.

P1 - “Ek doosre ko share kiya toh unko dilasa, mil jaega, atmabal baddjaega. Aur kuch seekne ko milega” [If we keep sharing with each other, it will help motivate us and we can learn from each other].

Financial constraints

In our study, most of the respondents were able to manage their healthcare finances. One participant mentioned that the medication had been expensive for him, while another claimed that in order to cover the costs, his spouse had to start working. Another participant mentioned that he resumed working again to relieve his family of the financial load caused by the cost of medicines.

P2 - “Zyada paisa dawa goli mein lag rahe hai, sugar aur BP ka takleef hai, heart ka goli hai, ek tube toh Rs 150 ka aata tha” [Most of the money is spent on medication, I have diabetes, BP and heart problem. One ointment cost around 150 INR].

P2 - “I have stopped going to work after surgery, now my wife goes for work.”

P3 - “Doosre ke khande pe nahi reh sakta, main goli ko, 2 paisa khud se kama kar” [I don’t want to be a burden on anyone to buy my medications, hence I have started working again].

Emotions surrounding CABG

Open-heart surgery is an ordeal that threatens several aspects of the patients’ and their families’ lives. The incapacity of the patients to perform their prior duties and responsibilities within the family or community can create fear and anxiety. One participant felt fearful and restless occasionally. Another young participant responded although there was stress initially, he had made peace.

P3 - “Tension kaay ghezach he anchya gharamadhle teesre operation aahe, manaja, behavicha operation” [Why should I be stressed after CABG? This is the third CABG surgery in my home, my maternal uncle and sister both underwent it].

P5 - “I am afraid and restless now. I feel lightheaded at times.”

P6 - “There have been stressful conditions at home after my CABG as I am young, 27 years old.” “Ho gaya toh ho gaya, abhi kya kar sakte hai” [Whatever has happened now has happened, what can we do?]. “I have become calmer than before.”

## Discussion

CABG surgery improves a patient’s survival rate, reduces the risk of angina, and increases their capacity for physical activities. On the other hand, CABG patients can also remain at high risk of coronary artery disease. Following surgery, secondary preventive measures such as self-management and cardiac rehabilitation are required to increase longevity and reduce future heart issues and the need for additional procedures. Self-management is an essential component of healing and that includes adopting or improving heart-healthy behaviours such as improving the quality of nutrition and increasing physical activity. Cardiac rehabilitation involves a variety of changes in lifestyle, especially through diet and exercise, regular follow-ups in the OPD, and implementing secondary preventive measures and recommendations. Participation in these programs is linked to a 20% reduction rate in cardiovascular mortality and morbidity, and consequently fewer medical expenses [3].

The utilisation and implementation of cardiac rehabilitation is limited by difficulties faced at both the provider and patient levels, which have been adequately described in Western literature [4].

Cardiac rehabilitation (CR) is underutilised, especially in regions with limited resources.

Ragupathi et al. found in their study that affordability, transportation challenges, primarily long distances to CR centres, a reluctance to participate in CR, and competing demands on patients’ time were all difficulties faced by patients [5].

Rashidi et al. discovered facilitators and impediments to treatment plan adherence among cardiovascular disease patients. Facilitators found were physical activity, support and mentorship, lifestyle modification, and the perceived benefits of medication. A perceived lack of support, reservations about taking medication, and a lack of engagement in exercise and lifestyle improvements were recognised as obstacles [6]. Overall, participants’ views of CR were favourable. The findings of this research support existing cardiac rehabilitation studies.

Dietary modifications

One of the integral parts of CR involves dietary modifications. It requires the consumption of a well-balanced meal with moderation in spices and restrictions of fat intake. In our study, most of the participants adjusted well to the new dietary requirements. Only one of them struggled in adapting to it.

Kalantarzadeh et al. observed in their qualitative study that participants’ preference for unhealthy cultural behaviours and beliefs were one of the barriers impacting treatment recommendations and lifestyle adjustments [7].

The qualitative study by Fix and Bokhour revealed that adhering to food restrictions was challenging [8]. This is consistent with the findings of our study.

Exercise

It was found that most of the participants were aware of the importance of exercise in their rehabilitation program while a few of them reported some reluctance in continuing the exercise regimens for life.

Back et al. found that patients who wished to live in the present would find it challenging to participate in an exercise-based rehabilitation programme. These patients did not value their participation in exercise-based CR as they wished to avoid worrying about the future. They also discovered that informants with physically demanding jobs viewed exercise as part of the job and did not believe that additional exercise in their leisure time was necessary [9].

A study by Mcintosh et al. found that fear of exercise acted as a barrier for cardiac rehabilitation therapy [10].

A study by Supervia et al. identified that one of the barriers in cardiac rehabilitation is anxiety in relation to exercise apart from finding transportation as a barrier [11].

Similar findings were reported by Pourhabib et al. People who attributed lifestyle factors to their heart illnesses were more inclined to continue with behavioural adjustments [12].

Addiction

Cigarette smoking is a predisposing factor for cardiovascular diseases. Few participants in our study couldn’t quit smoking, in line with the findings of the qualitative study by Kalantarzadeh et al. [7].

Support from CR team

In contrast to Kalantarzadeh et al.’s findings that concluded failure to offer follow-up assistance and continued contact with healthcare professionals after discharge were among the hurdles to treatment plan adherence [7], our study indicated that the CR team provided excellent healthcare service support.

Our findings were consistent with those of Rashidi et al., which also identified social support from health professionals and/or family as a crucial element in promoting treatment plan adherence [6].

Our results concurred with those of Nascimento et al. where participants thought that involvement in CR had long-term beneficial effects on their disease-related knowledge, increased functional gains, and enhanced psychosocial well-being [13]. Our results are consistent with those of the study conducted by McIntosh et al. [10].

Financial constraints and barriers to regular follow-ups

Financial burden remains one of the challenges for regular follow-ups in the CR programme.

It was found that cost of medication was one of the barriers to rehabilitation, which was similar to Kalantarzadeh et al.’s study [7].

Servio et al. found in their study that in comparison to non-participants, CR participants considered longer travelling distance, comorbidities, wait periods, and a lack of programme follow-up as barriers (the travel/work conflict subscale was significantly higher in this group) [14]. These findings were not reported by our participants during the in-depth interviews.

A study by Rangel-Cubillos et al. found that expenses associated with transportation were a significant barrier to participation in CR. They identified transportation cost as one of the greatest barriers of CR [15]. This finding was identical to our findings.

The results of this study demonstrated that challenges for cardiovascular patients to adhere to CR are complex and unlikely to be resolved by a single, focused intervention. Acknowledging patients’ limits, recognising the difficulties of altering lifestyles and beliefs and understanding the constraints of socio-cultural contexts can help policymakers, healthcare providers, especially nurses, establish tailored care plans and treatments suitable to the patients. Additionally, individuals with CVD should adhere to their CR regimen for an improved quality of life.

## Conclusions

The study revealed that it was challenging for cardiovascular patients to adhere to dietary restrictions. Controlling addictions was another issue, and transportation costs presented a challenge for CR follow-ups. However, the majority of the participants believed that, despite the challenges, CABG was a major procedure that motivated them to attend routine CR follow-ups and adhere to the CR regimen. All participants seemed satisfied when asked about the CR team’s behaviour, readiness to help, and motivation provided by them to continue following the CR regimens (diet, physical activity, follow-ups, etc.).

One of the strengths of this study was the presence of a good CR team with a protocol of telephonic reminders regarding follow-ups which led to a minimal drop-out rate.

## Appendices

**Table 2:** Interview Guide CR: Cardiac rehabilitation; CABG: Coronary artery bypass grafting.

Serial No.	Interview Guide
1.	Awareness regarding CR
2.	Importance of CR
3.	The doctor informed you regarding CR and explained the importance of adherence
4.	Problems faced in attending the CR follow up
5.	Advice given by doctors during discharge
6.	Management of time to follow the CR regimen
7.	Dietary Changes-burden or time-consuming
8.	Stress after CABG
9.	Experiences with CR team
10.	Need of a CR group
11.	Controlling addictions
12.	Exercise and behavioural change
13.	You can influence your own body and improve the existing condition without CR
14.	Impact of CR adherence on health in the long run

## References

[1] Khan MA, Hashim MJ, Mustafa H (2020). Global epidemiology of ischemic heart disease: results from the global burden of disease study. Cureus.

[2] Mehra VM, Gaalema DE, Pakosh M, Grace SL (2020). Systematic review of cardiac rehabilitation guidelines: quality and scope. Eur J Prev Cardiol.

[3] Kabboul NN, Tomlinson G, Francis TA (2018). Comparative effectiveness of the core components of cardiac rehabilitation on mortality and morbidity: a systematic review and network meta-analysis. J Clin Med.

[4] Clark AM, King-Shier KM, Spaling MA (2013). Factors influencing participation in cardiac rehabilitation programmes after referral and initial attendance: qualitative systematic review and meta-synthesis. Clin Rehabil.

[5] Ragupathi L, Stribling J, Yakunina Y, Fuster V, McLaughlin MA, Vedanthan R (2017). Availability, use, and barriers to cardiac rehabilitation in LMIC. Glob Heart.

[6] Rashidi A, Kaistha P, Whitehead L, Robinson S (2020). Factors that influence adherence to treatment plans amongst people living with cardiovascular disease: a review of published qualitative research studies. Int J Nurs Stud.

[7] Kalantarzadeh M, Yousefi H, Alavi M, Maghsoudi J (2022). Adherence barriers to treatment of patients with cardiovascular diseases: a qualitative study. Iran J Nurs Midwifery Res.

[8] Fix GM, Bokhour BG (2012). Understanding the context of patient experiences in order to explore adherence to secondary prevention guidelines after heart surgery. Chronic Illn.

[9] Bäck M, Öberg B, Krevers B (2017). Important aspects in relation to patients' attendance at exercise-based cardiac rehabilitation - facilitators, barriers and physiotherapist's role: a qualitative study. BMC Cardiovasc Disord.

[10] McIntosh N, Fix GM, Allsup K, Charns M, McDannold S, Manning K, Forman DE (2017). A qualitative study of participation in cardiac rehabilitation programs in an integrated health care system. Mil Med.

[11] Supervia M, Medina-Inojosa J, Martinez-Jarreta B, Lopez-Jimenez F, Vickers K, Terzic CM, Thomas RJ (2022). Cardiac rehabilitation completion study: barriers and potential solutions. J Cardiopulm Rehabil Prev.

[12] Pourhabib A, Sabzi Z, Yazdi K, Fotokian Z, Riahi Nokande GA (2022). Facilitators and barriers to return to work in patients after heart surgery. J Educ Health Promot.

[13] Nascimento IO, Assis MG, Ghisi GL, Britto RR (2021). A qualitative study of patient's perceptions of two cardiac rehabilitation models. Braz J Phys Ther.

[14] Sérvio TC, Britto RR, de Melo Ghisi GL (2019). Barriers to cardiac rehabilitation delivery in a low-resource setting from the perspective of healthcare administrators, rehabilitation providers, and cardiac patients. BMC Health Serv Res.

[15] Rangel-Cubillos DM, Vega-Silva AV, Corzo-Vargas YF (2022). Examining facilitators and barriers to cardiac rehabilitation adherence in a low-resource setting in Latin America from multiple perspectives. Int J Environ Res Public Health.

